# How control and eradication of BVDV at farm level influences the occurrence of calf diseases and antimicrobial usage during the first six months of calf rearing

**DOI:** 10.1186/s13620-024-00279-8

**Published:** 2024-09-28

**Authors:** Attila Dobos, Vilmos Dobos, István Kiss

**Affiliations:** 1 Ceva-Phylaxia Veterinary Biologicals Co. Ltd, Szállás u. 5, Budapest, H-1107 Hungary; 2 University of Veterinary Medicine, István u. 2, Budapest, H-1078 Hungary; 3 Large Animal Clinical Laboratory, Faculty of Veterinary Medicine, University of Veterinary Sciences, Brno, Czech Republic

**Keywords:** BVDV, Antibiotic usage, Calf mortality, Dairy cattle

## Abstract

**Background:**

Bovine viral diarrhoea (BVD) is one of the major cattle diseases causing economic losses worldwide. Nowadays the disease manifests mainly as virus-induced immunosuppression and early embryonic death, impacting overall herd performance and contributing to increased antibiotic usage in calf rearing.

**Methods:**

In our study we investigated the effect of rapid BVDV control measures on calf diseases and antimicrobial usage after weaning on a large industrial dairy farm. Persistently infected (PI) animals were identified and removed from the herd within a short period of time, and all susceptible animals were vaccinated against BVDV. Recorded herd parameters and AB usage were monitored retrospectively and compared with data collected after starting the BVD control program.

**Results and discussion:**

The programme began in January 2023 with identifying and eliminating PI animals from the farm. Twenty-one PI animals were found by using RT-qPCR testing of blood sera out of the 1571 animals tested (1.33%). Subsequent testing (January and December 2023) identified further 28 PI animals amongst the 542 calves tested shortly after birth, and all were instantly removed from the farm. In parallel with the BVDV eradication measures, AB usage dropped by more than 50% compared to previous years. Calf mortality also decreased from 7.45 to 4.38% as the control program progressed. Correspondingly, both the number of respiratory and diarrhoea cases decreased dramatically on the farm while the eradication measures were in place.

**Conclusion:**

Our study clearly demonstrated the positive effects of BVDV eradication on the improvement of calf health and importantly, a reduction of AB usage, contributing to the One Health perspective of farm animal production.

## Background

Bovine viral diarrhoea viruses (BVDVs) cause significant economic losses in dairy cattle farms worldwide, albeit acute BVD outbreaks are rare across Europe nowadays due to successful control programs [1, 2]. The cornerstones of BVD epidemiology are the so-called persistently infected immunotolerant animals, which emerge from dams naïve to BVDV that had gotten infected by a non-cytopathogenic biotype of the virus during the first trimester of gestation. Due to this early infection, and if survived that, such calves will be born and remain tolerant lifelong for the infecting virus strain and serve as primary reservoirs and sources of BVDV to their environment [1]. BVDV infections my remain unrecognised or overlooked because of so-called farm blindness [3], and only the deteriorating production parameters indicate the presence of the virus. Beyond that, the increasing number of respiratory problems or enteric diseases may raise the suspicion of an underlying BVDV infection. The virus causes immune dysfunctions, which leads to subsequent secondary bacterial infections and consequently, increasing use of antimicrobials on cattle farms [4]. Furthermore, BVDV infections readily contribute to Bovine Respiratory Disease Complex (BRDC) due to the immunosuppressive effects of the virus [5–9]. Respiratory infections represent a significant health concern and economic burden in calf populations, with their prevalence and severity on the rise globally. The frequency and severity of these infections are influenced by a range of factors, including the overall health status of the animals, their immune response, the quality of the administered medication, particularly the use of antibiotics, and the transmission of infectious agents [10–12]. Infectious diarrhoea represents a significant health concern in calves, accounting for over 50% of pre-weaned heifer calf mortality attributed to perceived causes in dairy herds [13]. Antibiotics are employed in the dairy industry for the treatment of a number of conditions, including mastitis, respiratory illness, lameness and enteric diseases. The two most prevalent diseases in dairy calves are calf diarrhoea and respiratory disease, with antibiotic treatments frequently employed in the treatment of these conditions [14, 15].

The objective of this study was to investigate the impact of control and eradication of BVDV at the farm level on the incidence of calf diseases and the use of antimicrobials during the initial six months of calf rearing.

## Materials and methods

Herd history. In January 2023, a major industrial dairy farm initiated a programme for the control of bovine viral diarrhoea virus (BVDV). The total number of Holstein Friesian cattle in the herd was 1,571, comprising 850 milking cows and 721 heifers. Prior to the investigation period, the herd had not been vaccinated against BVDV. Furthermore, no animals were introduced to the farm; instead, replacement stock was sourced from within the existing herd.

Sampling. All animals, 1571 capita, were blood sampled by jugular venipuncture at once for screening for the presence of suspected PI individuals. Subsequently, a biweekly sampling of newborn calves (< 4 weeks of age) was practiced over a period of one year.

A total of 2083 blood samples (1541 cows and heifers and all 542 newborn calves) were submitted for RT-qPCR and ab-ELISA tests.

Methods. A commercially available qPCR kit was used for screening to identify PI animals as described earlier [16]. In this case 25 samples were pooled, positive pooles split in to two groups (13 + 12), and if positive, items of these groups were further tested individually. The nucleotide sequences of the partial Npro coding genomic region was used for genotyping of the detected viruses according to Booth et al. [17]. Serological investigations were carried out by using the IDEXX BVDV Total Ab ELISA kit (IDEXX, USA) and by virus neutralization (VN) test, the latter to assess vaccine efficacy against the prevailing virus [16, 18, 19], using vaccine induced positive sera obtained from another herd.

### Disease definition and metrics

Individuals were considered PI animals with high viral load (Ct values < 27) of their respective serum samples in the general BVDV detecting qPCR. All virus positive animals with Ct values above 27 were retested four weeks later and if the calf remained PCR positive, it was considered to be PI.

Cases were identified, treated, and recorded in accordance with farm-specific criteria. These cases were identified and recorded by farmers and reflect their perceptions and ability to detect disease on a well-managed, large industrial farm.

The data pertaining to herd parameters were collated from the herd-management programme over the course of a four-year period, from January 2019 to December 2023. The investigation encompassed the occurrence of calf diseases and cases, as well as the administration of antibiotics to calves, and was conducted between the time of birth and the age of six months. The pertinent data from the farm are presented in tabular form in Table 1. Table 2 presents a detailed account of antimicrobial usage on the dairy farm during the initial six months of calf rearing, categorised by active substance and indication.

**Table 1:** Number of cows, calves born and calf mortality between 2019 and 2023

	2019	2020	2021	2022	2023
No of cows	782	823	805	847	842
No of calves born	774	797	826	845	843
No of mortality until 6 months of age	44	57	57	63	37
Calf mortality rate	5.68%	7.17%	7.62%	7.45%	4.38%

**Table 2:** Antimicrobial usage during the first six months of calf rearing by indication and active substance on the farm

Active substance	Indication of Antibiotic usage
Sulfadoxine/trimethoprim	Gastrointestinal infections
Florfenicol/Flunixin	Respiratory infections
Florfenicol	Respiratory infections
Tulathromycin	Respiratory infections, Metaphylaxis
Lincomycin/spectinomycin	Urinary infections, sepsis, Metaphylaxis
Benzyl penicillin/dihydrostreptomycin	Respiratory and Gastrointestinal infections
Oxitetracycline	Respiratory infections
Enrofloxacin	Respiratory and Gastrointestinal infections
Ceftiofur	Skin and soft tissue infections

## Results

The initial laboratory-confirmed detection of BVDV on the farm was in September 2020. Following the decision of farm management, the rapid eradication programme commenced on 13 January 2023.

Following the foundational steps of eradication, first the BVDV status of the entire herd (*n* = 1571) was assessed through investigating blood serum samples by virus specific RT-qPCR. This resulted in the identification of 21 supposedly PI animals. Their age distribution was as follows: 11 animals were 10–11 months old, 4 animals 7–8 months old and 6 calves 4–5 months old. These animals were promptly removed from the farm, thereby eliminating the direct sources of viral infection, and reducing the chance of further spread. Simultaneously, the farm began using a live, attenuated BVDV vaccine (Mucosiffa, Ceva-Sante Animale, France), applied concurrently to the entire herd - including all calves older than three months of age, in order to provide rapid and effective foetal protection for pregnant animals.

**Fig. 1:**
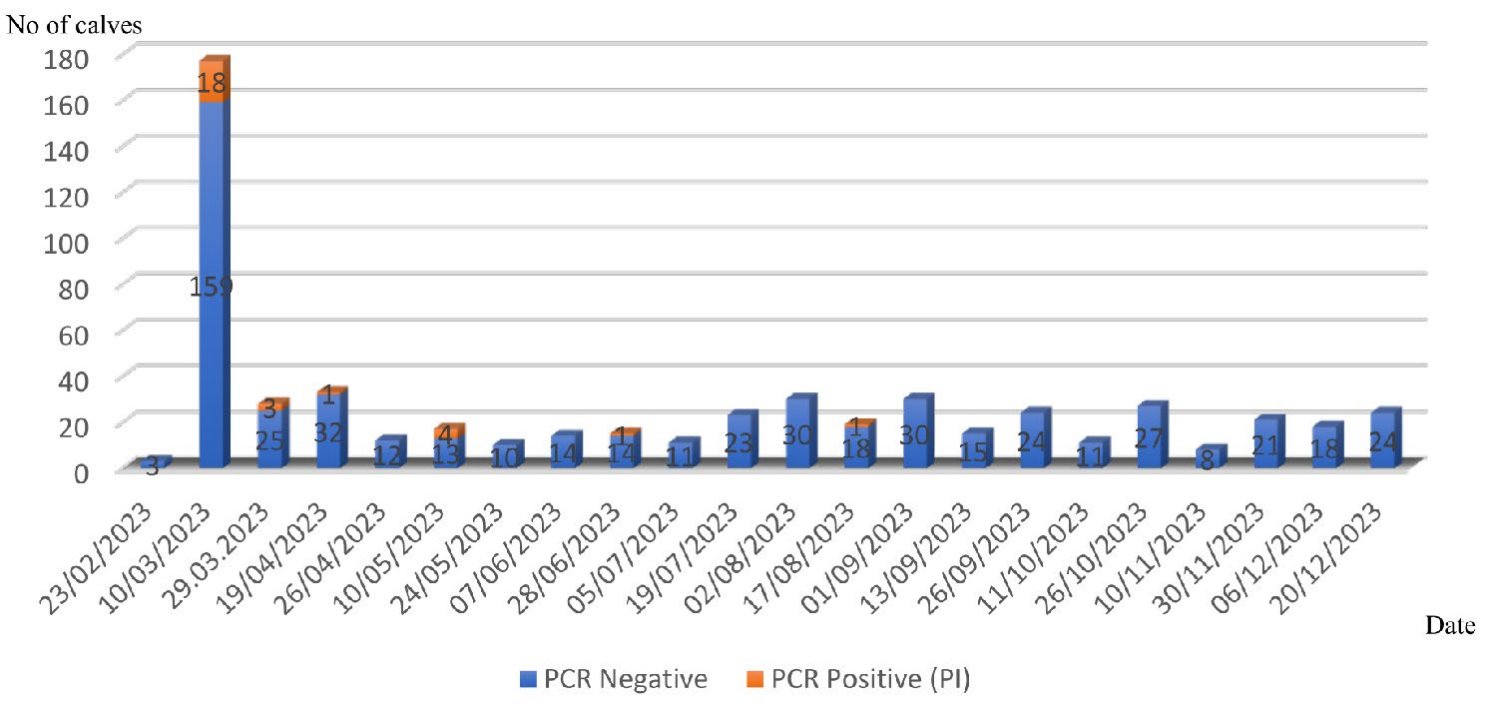
Sequential presentation of the number of newborn calves’ samples tested and of PI animals identified between 23/02/2023-20/12/2024 on the farm

After the initial phase of the programme, 542 newborn calves (< 4 weeks of age) were tested between the period of January and December 2023, which revealed 28 (5.16%) additional PI animals that were also removed from the herd (Fig. 1). The last recognized PI calf was born on 12th July 2023 (detected on 17th August 2023). The dam was 105 days pregnant when it was vaccinated. No further PI calves were found after this case.

All detected virus strains were classified as Pestivrius A subtype 1b. The VN measurements indicated that the vaccine induced antibodies were capable of neutralizing the viruses prevalent in the farm well beyond the suggested protective level, i.e. 1:20 [20].

The incidence of respiratory diseases, diarrhoea, lightweights and weak calves, and other diseases (urinary infections, skin and soft tissue infections, etc.) was recorded between 2019 and 2023 and is presented in Table 3, which compares and summarises the data.

Antimicrobials were primarily employed for the treatment of calves; however, tulathromycin was also frequently utilized for metaphylaxis in respiratory diseases. Lincomycin/spectinomycin constituted the second most frequently used antimicrobial combination, being administered to the majority of cases of sepsis and less frequently to other diseases, as well as being used for metaphylaxis. The most frequently administered antimicrobial combination in the case of respiratory diseases was enrofloxacin and florfenicol (also in combination with flunixin). Antimicrobials from other classes were employed for the treatment of diarrhoea and mild respiratory conditions. A detailed account of the number of antimicrobial treatments administered to calves is provided in Table 4.

**Table 3:** Occurrence of calf diseases and cases according to farm report

Calf Disease/Cases	2019	2020	2021	2022	2023
Respiratory disease	21	67	84	89	16
Diarrhoea	12	14	42	40	13
Weak calves	0	10	14	15	15
Other	22	14	18	25	24
Total	43	105	158	169	68

**Table 4:** Descriptive information about the number of antimicrobial treatments in calves between 2019 and 2023

Antimicrobial Treatment	2019	2020	2021	2022	2023
Sulfadoxine/trimethoprim	280	109	63	47	0
Florfenicol/flunixin	45	107	143	150	28
Florfenicol	0	106	112	110	0
Tulathromycin	320	520	570	634	230
Lincomycin/spectinomycin	280	242	238	218	179
Benzyl penicillin/dihydrostreptomycin	30	5	16	16	4
Oxitetracycline	57	0	56	24	14
Enrofloxacin	0	105	185	193	124
Ceftiofur	0	0	24	30	20
Total Treatments	1012	1194	1407	1422	599

## Discussion

As dairy herds have expanded and modernized, the focus of health management has shifted from treatment to prevention over the last twenty years [21]. Today, BVD is recognised as one of the most economically important endemic diseases of cattle [22]. Outbreaks of virulent BVDV are rare while other manifestations of the virus infection are becoming increasingly common. In calves BVDV have mainly been associated with pneumonia and enteritis [5, 23, 24]. A significant relationship was found between the BVDV infection status of herds and the incidence of calf mortality and respiratory disorders [6, 25]. Studies also indicated that BVDV plays an important role in enteric diseases when occurring in conjunction with other enteric pathogens. For example, concurrent infection with BVDV and bovine rotavirus (BRV) causes more serious enteric disease than BRV infection alone [26].

The use of antibiotics represents a significant concern in the field of animal husbandry. For instance, the overuse of antibiotics may result in an increased prevalence of bacterial resistance, which is a crucial issue with implications for human healthcare. A significant component of the strategy to curtail the utilisation of antibiotics is the implementation of appropriate prophylactic measures, such as targeted vaccination and enhanced biosecurity protocols. These strategies are instrumental in reducing the incidence of antibiotic use, whether directly, for the treatment of primary infections predominantly caused by viruses, or indirectly, for the management of secondary infections predominantly caused by bacteria.

Most of the antimicrobial use in dairy calves is related to respiratory and enteric diseases [15], which coincides with our results regarding the follow-up of antimicrobial usage during BVDV eradication.

Demonstrating the presence of BVDV on the farm greatly contributed to the farmer’s supposition that there had to be an underlying factor behind the lower-than-expected herd performance. The detected 1b strain confirms previous recent findings on the prevalence of BVDV subgenotypes in the country, i.e. 1b, 1d, and 1f [16]. The identification of PI animals was based on the viral load in blood, which had been reported an appropriate tool for the purpose and was based on fact that observed values are significantly higher in PI compared to transiently infected (TI) animals [27]. Using the highly sensitive RT-qPCR approach enabled pooling of the samples without risking detection sensitivity. Nevertheless, re-testing suspected PI animals in due time (3–4 weeks apart) would have been more accurate, but the farm management did not want to risk further spreading of the virus and decided to embark on rapid elimination of such individuals.

Prior to the detection of BVDV on the farm (2019), the mortality rate of calves was 5.68%. The leading causes of mortality between birth and six months of age were respiratory disorders and diarrhoea. Over the course of the following three years (2020, 2021 and 2022), there was a marked increase in the annual mortality rate of calves, reaching 7.17%, 7.62% and 7.45% respectively. In the vast majority of cases (98%), the underlying cause was disease, with respiratory disorders emerging as the primary cause of mortality, accounting for approximately 60% of deaths in each year. In 2023 - when the BVDV control measures started - calf mortality was 4.38%. Throughout the BVDV control program (2023), 37 calves died before six months of age but less than 30% of these cases were due to respiratory illness.

**Fig. 2:**
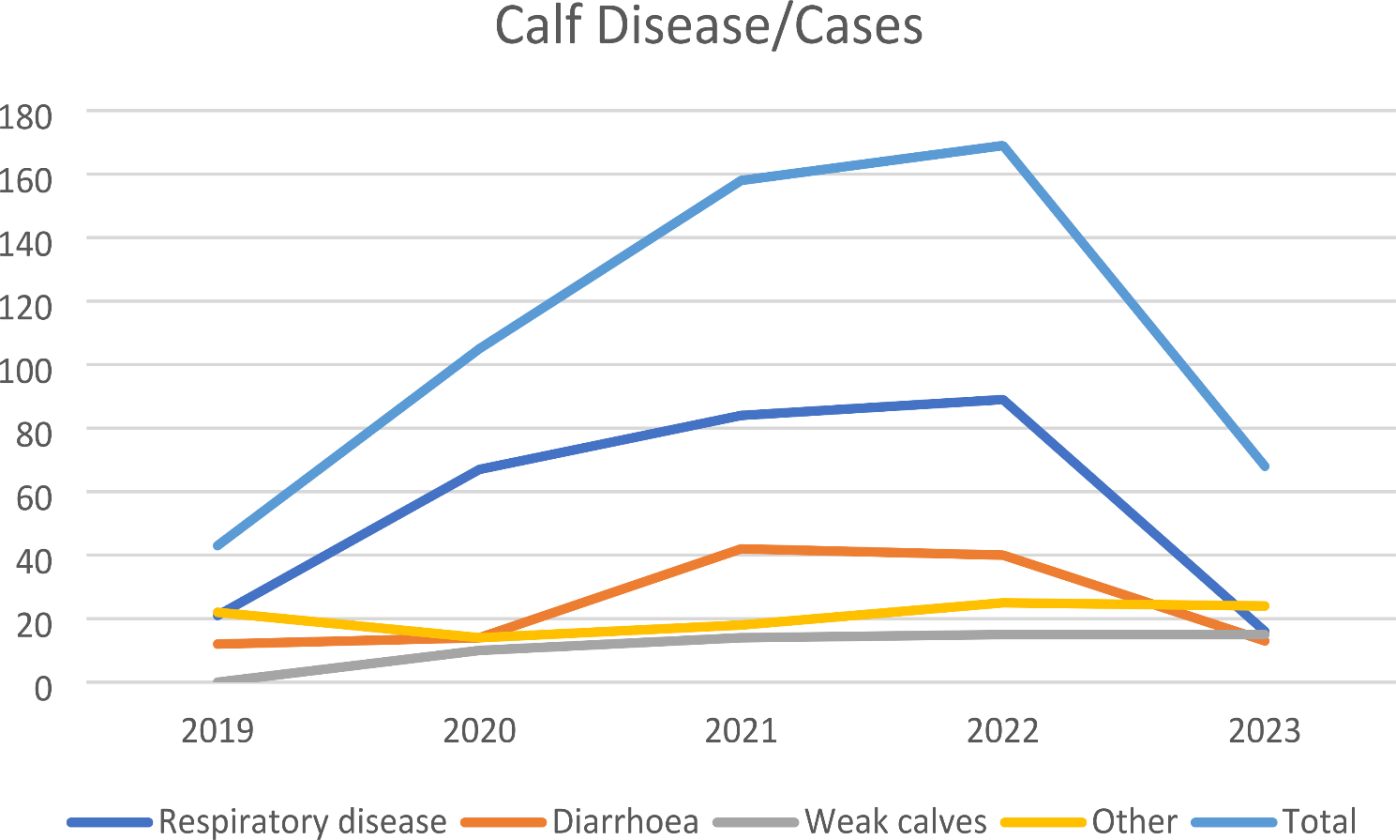
Trends regarding the various calf diseases and the number of cases for each disease between 2019 and 2023

The incidence of calf diseases has demonstrated a similar trend to that observed in calf mortality (Table 4). Prior to the detection of the BVD virus, 21 out of 43 animals (48%) exhibited respiratory issues, while 12 out of 43 calves (28%) displayed diarrhoea. Over the subsequent three-year period, the number of cases of respiratory disease increased threefold in comparison to the previous year. Similarly, the incidence of diarrhoea exhibited a parallel pattern. A new category was introduced to the herd management program, designated as “weak calves,” which likely encompasses the PI animals. The implementation of the BVDV control programme resulted in a 71% reduction in the incidence of respiratory disorders and a 67.5% reduction in the incidence of diarrhoea. However, the weak calves that had been identified in previous years were classified as PI animals and were promptly removed from the farm. Figure 2 illustrates the trends in the incidence of calf diseases and the number of cases for each disease between 2019 and 2023.

**Fig. 3:**
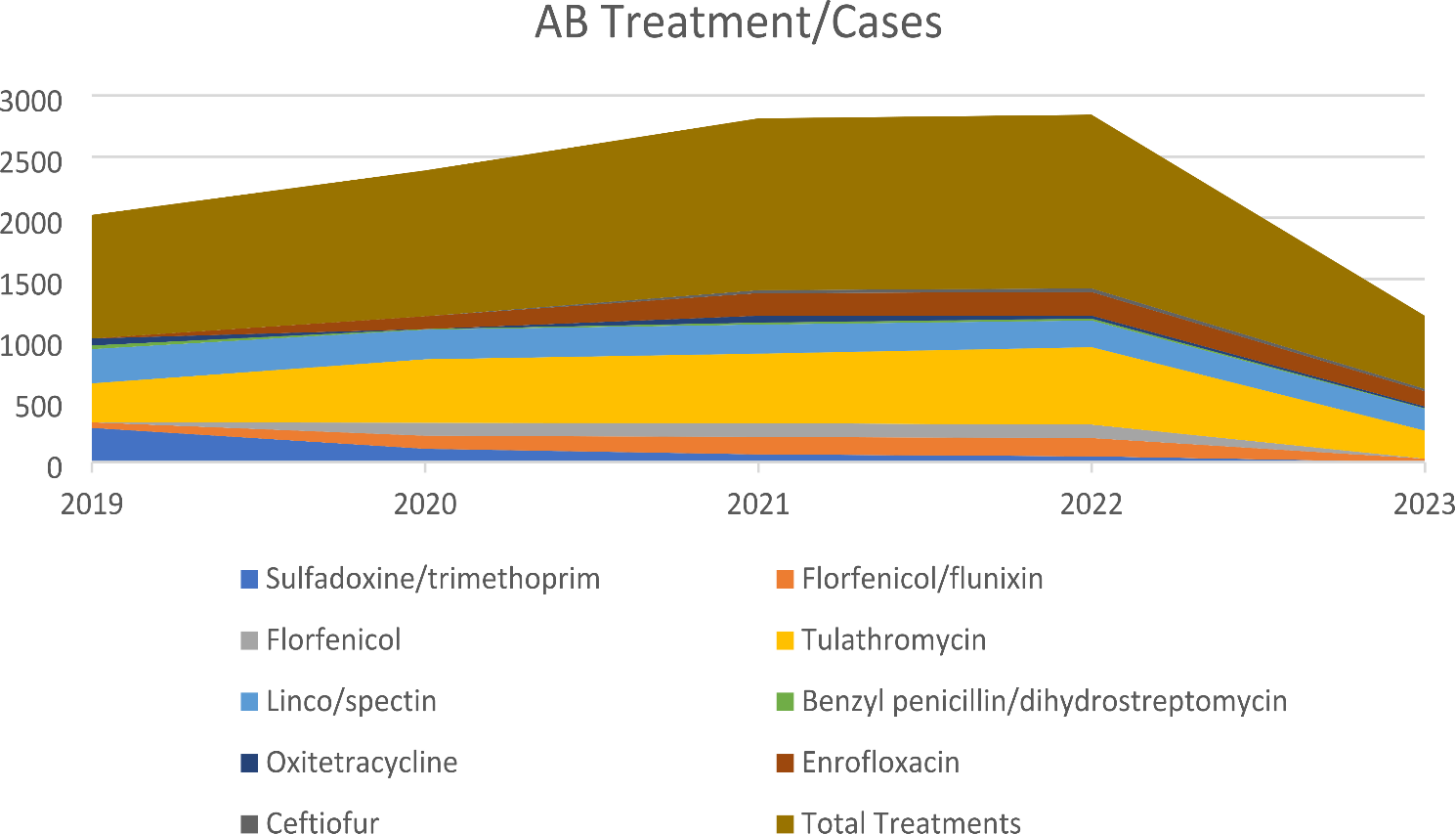
Number of AB treatments according to active substances between 2019 and 2023

In 2019, a total of 1,012 cases of antibiotic treatment were recorded on the dairy farm prior to the initial detection of BVDV. Over the subsequent three-year period, there was a marked increase in the usage of antimicrobial agents. The total number of antimicrobial treatments administered during these years was 1,194, 1,407, and 1,422, respectively. During the course of the BVDV eradication programme, there was a marked decline in the utilisation of antimicrobial agents. The number of antibiotic treatments was reduced to less than 600, representing a decrease of over 57.9% compared to the previous year. The reduction in antimicrobial usage was observed across all active ingredients, with the greatest decline observed in those associated with calf respiratory diseases, namely tulathromycin and florfenicol (Fig. 3). Tulathromycin is a widely used antimicrobial agent for the metaphylaxis of bovine respiratory diseases across the globe, predominantly in feedlot cattle but also in the dairy industry. The findings of our study suggest that a greater emphasis on the prevention and/or eradication of infectious diseases such as BVDV could prove an effective strategy for reducing AB usage. Given the immunosuppressive nature of BVDV, such control programmes will permit the treatment of only those animals that are sick. The metaphylactic treatment of healthy animals may therefore be unnecessary in order to prevent the further spread of secondary infectious diseases.

## Conclusion

The results of our study clearly demonstrate the positive effects of BVDV eradication. In addition to eliminating the virus and reducing its direct impacts, it has also led to improvements in calf health. Furthermore, it has contributed to a reduction in the use of antibiotics (ABs), which is a fundamental aspect of the One Health approach to farm animal production.

## Data Availability

The datasets used and analysed during the current study are available from the corresponding author on reasonable request.

## Abbreviations

AB: Antibiotic
BRDC: Bovine Respiratory Disease Complex
BRV: Bovine Rota Virus
BVD: Bovine Viral Diarrhoea
BVDV: Bovine Viral Diarrhoea Virus
ELISA: Enzyme-linked immunosorbent assay
PI: Persistently Infected Animal
RT-qPCR: Real-time polymerase chain reaction
TI: Transiently Infected Animal
VN: Virus neutralization
